# Heterostructures of Cut Carbon Nanotube-Filled Array of TiO_2_ Nanotubes for New Module of Photovoltaic Devices

**DOI:** 10.3390/nano12203604

**Published:** 2022-10-14

**Authors:** Siqi Niu, Wenbin Yang, Heng Wei, Michail Danilov, Ihor Rusetskyi, Ketul C. Popat, Yao Wang, Matt J. Kipper, Laurence A. Belfiore, Jianguo Tang

**Affiliations:** 1 Institute of Hybrid Materials, National Center of International Joint Research for Hybrid Materials Technology, National Base of International Sci. & Tech. Cooperation on Hybrid Materials, Qingdao University, 308 Ningxia Road, Qingdao 266071, China; 2 V.I. Vernadskii Institute of General and Inorganic Chemistry of the Ukrainian NAS, 32/34 Palladin avenue, 03142 Kyiv, Ukraine; 3 Department of Mechanical Engineering, Colorado State University, Fort Collins, CO 80523, USA; 4 Department of Chemical and Biological Engineering, Colorado State University, Fort Collins, CO 80523, USA

**Keywords:** TiO_2_ nanotubes, cut-MWCNTs, heterostructure, photovoltaic devices

## Abstract

In this work, a new photovoltaic device was prepared. The device uses titanium (Ti) foil/TiO_2_ nanotubes as the photoanode and multi-walled carbon nanotubes (MWCNTs) as a photosensitizer. Titanium dioxide nanotube arrays (TiO_2_-NTs) were prepared by one-step anodic oxidation. Cut-MWCNTs with a length of less than 100 nm were obtained by the mixed-acid oxidation of MWCNTs. The two materials were combined to form a TiO_2_-NTs@cut-MWCNT heterostructure by electrophoresis. TiO_2_-NTs@cut-MWCNTs were characterized by field-emission scanning electron microscopy (FESEM) and X-ray diffraction (XRD), which showed that the two materials were effectively combined. We fabricated the heterostructure into a photovoltaic device, showing an enhanced photocurrent response and an efficiency of 0.0138%, and explained this phenomenon by performing UV-vis absorption spectroscopy and electrochemical tests. It is hoped that this work can provide a reference value for the application of carbon nanotubes in photovoltaic devices.

## 1. Introduction

Solar energy is renewable energy with the advantages of wide distribution, easy access, and inexhaustible supply. As an effective way to utilize solar energy, solar cells have always been a research hotspot [1]. Silicon-based solar cells are the most mature at present, but they are expensive, consume lots of energy in the production process, and have a long cost recovery period, which are all barriers to their large-scale application and promotion. In 1986, Dr. C.W. Tang [2] first prepared a double-layer organic solar cell device with copper titanate cyanine as the donor material and perylene as the acceptor material and tested it under simulated sunlight to obtain an efficiency of 1%. In 1991, O’Regan and Grätzel [3] manufactured the first nanocrystalline TiO_2_ chemical solar cell. Compared with traditional solar cells, this cell has the advantages of a simple manufacturing process, low production cost, and non-toxic and pollution-free production, and it is expected to compete with silicon technology in the future [4]. In contrast, the light-absorbing layer materials of dye-sensitized solar cells and polymer solar cells need to undergo complex organic synthesis, and their stability is not high. Therefore, researchers have been exploring new materials, new processes, and new structures.

TiO_2_ is an excellent candidate semiconductor material for the effective use of solar energy because of its appropriate band gap, low cost, good chemical stability, and photostability. Since Grimes et al. [5] first reported the successful preparation of highly ordered TiO_2_ nanotube arrays (TiO_2_-NTs) by electrochemical anodization, the synthesis and application of one-dimensional tubular array structures represented by TiO_2_-NTs have increased rapidly. This structural material has a high orientation, can provide a good path for electron transfer, and is simple to manufacture. Its preparation avoids the cumbersome process of organic synthesis, provides the possibility for large-scale roll-to-roll production, and is proven to have great application value in photovoltaic devices [6,7,8,9,10,11].

Although TiO_2_ has high photoactivity among many oxide semiconductors, its ability to use sunlight is still very limited, and the recombination rate of photogenerated charges is high, resulting in low quantum yield. Therefore, a lot of work has been conducted to develop modifications of TiO_2_. Carbon nanotubes are cheap and easy to obtain by chemical treatment. Their energy-level position and band-gap width can be changed to match the energy level of TiO_2_. They can therefore be used as a photosensitizer “dye”. Multi-walled carbon nanotubes (MWCNTs) are composed of multiple single-walled carbon nanotubes (SWCNTs). The density of states of each single-walled carbon nanotube has Van Hove singularity and exhibits a photovoltaic effect. Therefore, they have attracted great attention in the field of photoelectrochemistry [12,13,14,15,16,17,18,19]. Kumar and colleagues [20] used TiO_2_ and MWCNTs as active charge-generating layers by spin-coating them on transparent conductive substrates to fabricate solar cells. The efficiency of the TiO_2_@MWCNT solar cell was 0.4%. Kalitag et al. [21] embedded cut MWCNTs into polymer films to improve their performance and found that cut MWCNTs have better hole transport ability. Baker et al. [22] reviewed carbon solar cells, which use carbon nanotubes as the photosensitive layer with semiconductor properties.

In this work, we propose an all-titanium–carbon photovoltaic device. TiO_2_-NTs were first prepared by anodization, cut-MWCNTs were prepared by mixed-acid oxidation, and then the two materials were combined to form a TiO_2_-NTs@cut-MWCNT heterostructure by a simple electrophoresis method [23]. Although the heterostructure formed by the composite of TiO_2_ and carbon nanotubes has been applied in the field of photocatalysis, its functional mechanism for light energy conversion has not yet been reported [24,25,26], and there is no example of fabricating photovoltaic devices using only TiO_2_ and carbon nanotubes. Therefore, we prepared an all-titanium–carbon photovoltaic device based on the TiO_2_-NTs@cut-MWCNT heterostructure. The charge-transfer mechanism in the device was studied by electrochemical tests. Most importantly, the material cost of the photovoltaic device is low, and the experimental method is simple. We hope this work can provide some reference value for the application of carbon nanotubes in photovoltaic devices.

## 2. Materials and Methods

### 2.1. Fabrication of TiO_2_-NTs

Pure Ti foil (150 µm thick, 99.99%) was sanded and then ultrasonically cleaned with isopropanol, ethanol, and deionized water for 10 min each. The natural surface oxides were then removed with hydrochloric acid (HCl:H_2_O = 1:1) [27]. The cleaned foils were stored in DI water for later use. Pt foil (100 µm thick, 99.9%) was used as the counter electrode. The electrolyte solution was 95 vol% diethylene glycol (DEG), 2 vol% HF, and 3 vol% DI water. Highly ordered TiO_2_-NTs were prepared by electrochemical anodization at 55 V for 1 h at room temperature. After anodization, the anodized TiO_2_ nanotube array samples were rinsed in isopropanol and DI water to remove the electrolyte and dried in a fume hood, followed by annealing at 530 °C for 3 h in an air atmosphere to obtain the crystal structure.

### 2.2. Cutting of the MWCNTs

MWCNTs (purity > 95%; length: 0.5–2 μm) were purchased from Macklin. The cutting steps are as follows: 100 mg of MWCNTs was placed in 30 mL of concentrated H_2_SO_4_ and stirred at room temperature for 30 min to obtain a uniform suspension. The suspension was then mixed with 18 mL of concentrated HNO_3_ and heated and stirred at 70~80 °C for 10 h. The cut carbon nanotubes were collected by centrifugation and washed repeatedly with DI water until the pH value reached neutrality.

### 2.3. Preparation of the Heterostructure of TiO_2_-NTs@cut-MWCNTs

The heterostructure of TiO_2_-NTs@cut-MWCNTs was formed by electrophoresis. Cut-MWCNTs were dispersed in deionized water and then sonicated to form a homogeneous suspension. The as-prepared TiO_2_-NTs were used as substrates. In the cut-MWCNT suspension, Pt foil used as the counter electrode was kept 1 cm parallel to the TiO_2_ electrode. Figure S1 is a schematic diagram of the electrophoresis device using an RPS6005C-2DC stabilized power supply. When a forward voltage was applied, the negatively charged cut-MWCNTs in the solution moved directionally into the TiO_2_-NTs under the action of the electric field. When a reverse voltage was applied, part of the cut-MWCNTs in the suspension reoriented and moved. When the forward voltage was reapplied, the moving direction of the cut-MWCNTs changed again. Alternating the sign of the voltage many times could improve the contact between cut-MWCNTs and TiO_2_-NTs. The electrophoresis voltage was 20 V, the forward voltage was applied for 30 s, and the reverse voltage was applied for 5 s; this cycle was repeated 30 times. Finally, the samples were rinsed with deionized water 3 times and dried overnight at 60 °C.

### 2.4. Fabrication of All-Titanium–Carbon Photovoltaic Device

**Figure 1:**
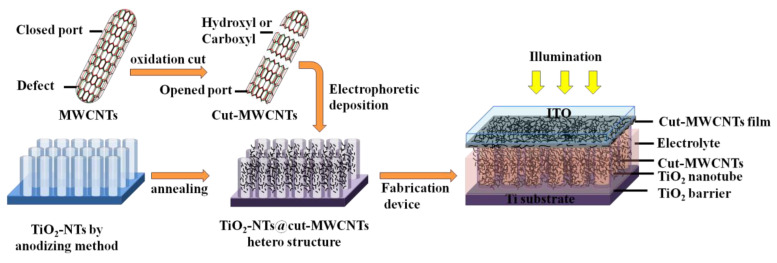
Schematic diagram of experimental process and device structure.

The photovoltaic device is mainly composed of carbon nanotubes and TiO_2_, so it is called an “all-titanium–carbon photovoltaic device”. The preparation process of the device is as follows: (1) Preparation of the photoanode: The prepared TiO_2_-NTs@cut-MWCNT heterostructure was used as the photoanode. (2) Preparation of the counter electrode: In order to avoid the short circuit of the direct contact between TiO_2_-NTs and ITO inside the device, ITO spin-coated with a small amount of cut-MWCNTs was used as the counter electrode. (3) Preparation of electrolyte: The electrolyte was a 0.2 M K_3_[Fe(CN)_6_]/K_4_[Fe(CN)_6_] aqueous solution. (4) Assembly of the device: The photoanode was placed in the sealed insulating groove and covered with the counter electrode, the electrodes were staggered and clamped with clips, and the electrolyte was injected from the edge. In order to avoid an internal short circuit, a few droplets were used to test the efficiency. The experimental process and device structure are shown in Figure 1.

### 2.5. Characterization

MWCNTs and cut-MWCNTs were characterized by TEM, FESEM, Raman, FT-IR, XRD, BET surface analysis, and XPS. TEM images were obtained with a Thermo Scientific F200i (USA) transmission electron microscope. FESEM images were obtained with a Regulus 8100. The Raman spectra were obtained using an Almega Thermo Nicolet Dispersion Raman spectrometer (laser wavelength: 532 nm). Fourier transform infrared spectroscopy (FT-IR) data were measured using a Nicolet 6700 Fourier transform infrared spectrometer from Thermo Corporation (Waltham, MA, USA). XRD was performed with a Bruker D8 Advance model. BET surface analysis was measured using Micromeritics ASAP 2460. XPS data were measured using the Thermo Scientific K-Alpha Spectrometer (USA). Optical properties were characterized by a UV-vis spectrometer (Lambda 750s) with a wavelength range of 200 to 800 nm.

TiO_2_-NTs and TiO_2_-NTs@cut-MWCNTs were analyzed by FESEM, UV-vis, and XRD. FESEM images were obtained on a Hitachi-SU8220 at an accelerating voltage of 20 kV without gold plating. The spectral absorptions of TiO_2_-NTs and TiO_2_-NTs@cut-MWCNTs were obtained using PerkinElmer Lambda 750s with an integrating sphere. The crystal structures of the surface were analyzed by X-ray diffraction (XRD) using a Bruker D8 Advance diffractometer (Co Kα radiation, λ = 1.54056 nm).

## 3. Results and Discussion

### 3.1. Characterizations of Cut-MWCNTs

**Figure 2:**
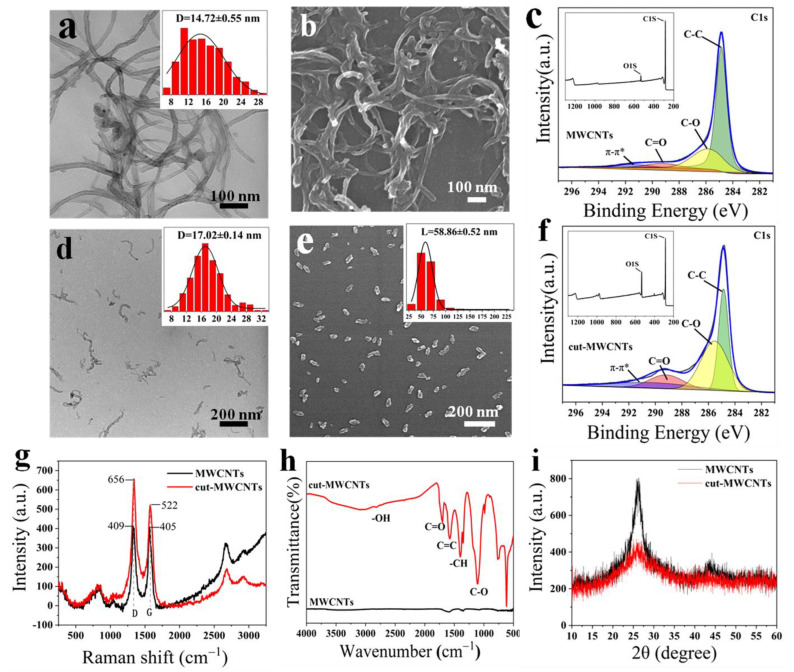
Pristine MWCNT morphology observed by (**a**) TEM and (**b**) FESEM; (**c**) XPS C1s spectrum of MWCNTs, with the survey scan shown in the inset; cut-MWCNT morphology observed by (**d**) TEM and (**e**) FESEM; (**f**) XPS C1s spectrum of cut-MWCNTs, with the survey scan shown in the inset; (**g**) Raman spectra of MWCNTs and cut-MWCNTs; (**h**) FT-IR spectra of MWCNTs and cut-MWCNTs; (**i**) XRD patterns of MWCNTs and cut-MWCNTs.

MWCNTs often contain high-density pentagonal and heptagonal defects on the sidewalls of the nanotubes [28]. During the mixed-acid oxidation process, these positions will be preferentially destroyed. Figure 2a,b show the TEM and FESEM images of MWCNTs, respectively. It can be seen that the length of MWCNTs is so long that the tubes are entangled with each other, and the average diameter of the MWCNTs is calculated to be 14.72 ± 0.55 nm (counted number: 500). Figure 2d,e show the TEM and FESEM images of cut-MWCNTs, respectively. It can be seen that the length of cut-MWCNTs becomes shorter, and the dispersibility becomes better. Due to the uneven distribution of defects on the surface of carbon nanotubes, the length distribution of cut-MWCNTs is uneven, the average length of cut-MWCNTs is 58.86 ± 0.52 nm (counted number: 500), and the average diameter is 17.02 ± 0.14 nm (counted number: 500). This is because, during the oxidation process, the walls of the carbon nanotubes are destroyed. By comparison, it can be concluded that the ratio of the length to the diameter of cut-MWCNTs is significantly smaller than that of MWCNTs.

The Raman spectra provide molecular vibration and rotation information. Through the analysis of the position and intensity of the characteristic peaks in the spectrum of MWCNTs and cut-MWCNTs, changes in the composition and structure can be discerned. Figure 2g shows the Raman spectra of MWCNTs and cut-MWCNTs. The spectra of MWCNTs and cut-MWCNTs contain D and G peaks. The D peak at 1345 cm^−1^ is related to the structural defects of MWCNTs, carbon nanoparticles adsorbed on the tube wall, amorphous carbon, and other factors, which are generally considered to be caused by the defects of MWCNTs. The G peak at 1592 cm^−1^ is due to the sp^2^ hybridization of carbon atoms. The ratio of the D peak to the G peak, R, is used to characterize the structure of carbon nanotubes. The R value of MWCNTs is 1.01, and the cut-MWCNTs have an increased R of 1.26, indicating that part of the ordered graphene structure was destroyed by the acid treatment used for cutting [29].

Mixed-acid treatment will introduce acidic groups to the surface of the carbon nanotubes [30]. Cut-MWCNTs were analyzed by infrared spectroscopy, and the types of functional groups were determined according to the positions of absorption peaks. Figure 2h shows the infrared spectra of MWCNTs and cut-MWCNTs. Compared with MWCNTs, cut-MWCNTs have a wide peak at 3500–2000 cm^−1^, which is caused by the stretching vibration of -OH, meaning that acid treatment can introduce -OH to the surface of cut-MWCNTs. The peak at 1707 cm^−1^ is associated with the C=O stretching of carboxylic acids. The absorption peak near 1580 cm^−1^ corresponds to the stretching vibration of the C=C bond, which is inherent in the structure of carbon nanotubes. The peaks at 1404 cm^−1^ and 1100 cm^−1^ are obviously enhanced, corresponding to the in-plane bending vibration of -CH and the stretching vibration of C-O, respectively. These absorption peaks indicate that there are a large number of oxygen-containing functional groups on the surface of cut-MWCNTs, which increase the negative surface charge and enhance the electrostatic repulsion between cut-MWCNTs. The strong electrostatic repulsion makes the dispersion of carbon nanotubes in solution more uniform and prevents aggregation. This is consistent with the TEM and FESEM results and is conducive to the deposition of cut-MWCNTs on the surface of TiO_2_-NTs by electrophoresis.

The XRD patterns reflect the crystalline structural features of MWCNTs and cut-MWCNTs, which were tested under the same conditions. As shown in Figure 2i, the peak at 26.1° is the diffraction peak of the graphitic layer structure (002), from which the crystalline structural changes in carbon nanotubes before and after cutting can be seen. The peak of pristine MWCNTs is sharper and stronger. However, the peak of cut-MWCNTs at 26.1° is broader and much lower, and the intensity drops significantly, indicating that the initial graphitic structure of MWCNTs is oxidized and has more amorphous features. A large number of oxygen-containing functional groups change the atomic arrangement of MWCNTs.

The surface properties of MWCNTs and cut-MWCNTs were studied by N_2_ adsorption analysis. As can be seen from the nitrogen (N_2_) adsorption–desorption isotherm in Figure S2a, the samples have obvious lag loops at relative pressures between 0.7 and 1.0, indicating the existence of mesopores. The carbon nanotubes show an increase in the surface area from 105 m^2^/g to 155 m^2^/g due to oxidative cutting. The increase in the specific surface area can be attributed to the combined effect of the unbundling of MWCNTs and the opening of end caps, leading to a higher uptake of nitrogen at a relative pressure of 0.7 to 1.0 in the cut-MWCNTs due to the increase in mesopores [31], while the length of cut-MWCNTs is shorter and can achieve better contact with TiO_2_-NTs. In addition, the slight N_2_ uptake at low pressures suggests the co-existence of micropores. The NL-DFT pore size distribution further verifies the pore structure, showing that the main pores are centered in a wide range of 1–10 nm (Figure S2b). It is interesting to find that there is a new fraction of pores at 3.4 nm for cut-MWCNTs, suggesting that pickling is beneficial to the formation of mesopores.

To further study the chemical bonding of MWCNTs in the oxidized state, we carried out XPS characterization of the MWCNTs and cut-MWCNTs. The spectra of C1s before and after oxidation (Figure 2c,f) can be divided into four peaks. The peak areas corresponding to C-O and C=O increased significantly after oxidation. Table 1 lists the elemental composition of MWCNTs and cut-MWCNTs, also showing a significant increase in the oxygen atom percentage after oxidation. MWCNTs can be regarded as coiled multilayer graphene. Mixed-acid oxidation consumes π-conjugated groups and introduces oxygen-containing functional groups between graphite layers [32], which is consistent with the FT-IR results. In addition, the contents of N and S are also increased slightly, but the remaining N and S functional groups have no mobility after the strong and repeated washing process, so they have no influence on the device efficiency.

### 3.2. Characterizations of TiO_2_-NTs@cut-MWCNT Heterostructure

**Figure 3:**
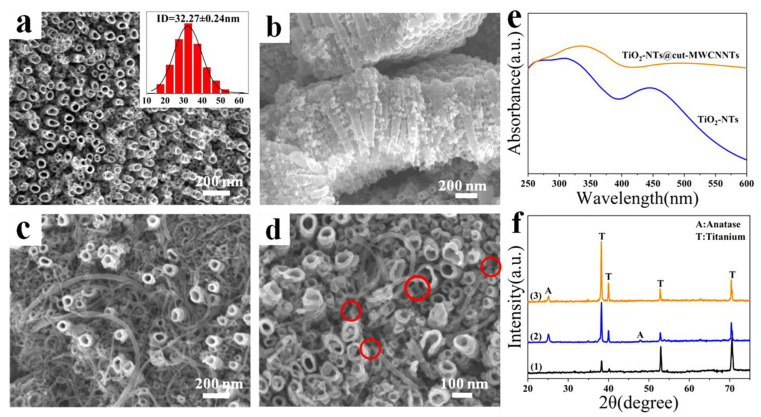
SEM images of TiO_2_-NTs (**a**,**b**) and TiO_2_-NTs@cut-MWCNTs (**c**,**d**); (**e**) UV-vis absorbance spectra of TiO_2_-NTs and TiO_2_-NTs@cut-MWCNTs; (**f**) XRD patterns of TiO_2_-NTs before (1) and after annealing (2) and the TiO_2_-NTs@cut-MWCNTs (3).

Figure 3a,b show SEM images of pure TiO_2_-NTs. The pure TiO_2_-NTs indicate a clear tubular structure. The average inner diameter is 32.27 ± 0.24 nm, and the tube lengths are about 600~700 nm. Figure 3c,d are SEM images of TiO_2_-NTs@cut-MWCNTs, in which the deposited cut-MWCNTs can be found in the pores and gaps among the TiO_2_-NTs, as marked in Figure 3d (Figure S3 is a cross-sectional SEM image of the TiO_2_-NTs@cut-MWCNT heterostructure).

To demonstrate the effect of cut-MWCNTs on the light absorption properties of TiO_2_-NTs, Figure 3e shows the UV-vis absorption spectra of TiO_2_-NTs and TiO_2_-NTs@cut-MWCNTs. Compared with TiO_2_-NTs, the light absorption of the TiO_2_-NTs@cut-MWCNT heterostructure significantly increased, and at the same time, its absorption edge was red-shifted from 310 nm to 336 nm, indicating that due to the recombination of cut-MWCNTs, the energy band of TiO_2_-NTs is narrower and is more readily excited by visible light.

XRD was employed to characterize the crystalline phase of the TiO_2_-NTs@cut-MWCNT heterostructure, as shown in Figure 3f. Before the annealing of TiO_2_-NTs, all peaks were attributed to the Ti substrate. After annealing, peaks appeared at 25.1° and 48°, corresponding to the (101) and (200) crystal planes of the anatase phase of TiO_2_, indicating that annealing changed the crystal form of TiO_2_ from the amorphous phase to the anatase phase. Anatase TiO_2_ is a relatively stable structure with high band-gap energy. There are more defects and vacancies in the lattice, resulting in more oxygen vacancies to capture electrons, so it has high activity. In the XRD patterns of the TiO_2_-NTs@cut-MWCNT heterostructure, the (101) plane (2θ = 25.1°) of the anatase phase can be seen. However, there are almost no diffraction peaks to be found for cut-MWCNTs. This is because the cut-MWCNTs have a broad and weak diffraction peak at 2θ = 26.1°, as shown in Figure 2i, indicating very weak crystalline characteristics. Thus, in the TiO_2_-NTs@cut-MWCNTs, the lower content of cut-MWCNTs results in a signal that is too weak to detect.

### 3.3. Characterizations of All-Titanium–Carbon Photovoltaic Device

**Figure 4:**
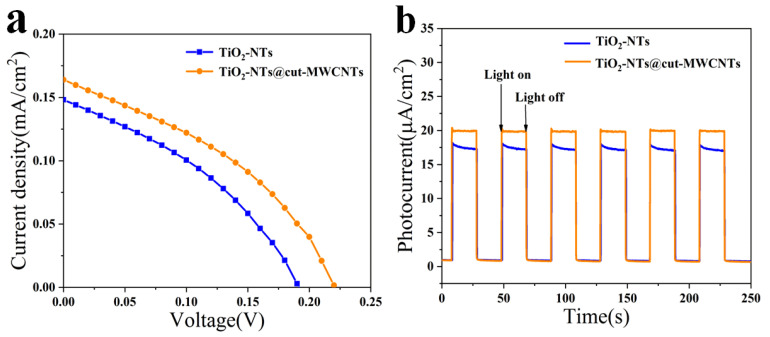
(**a**) J-V curves of devices based on TiO_2_-NTs and TiO_2_-NTs@cut-MWCNTs; (**b**) the transient photocurrent density under visible light and dark conditions for TiO_2_-NTs and TiO_2_-NTs@cut-MWCNTs.

The TiO_2_-NT and TiO_2_-NTs@cut-MWCNT heterostructures were each assembled into photovoltaic devices, and the current-density–voltage (J-V) characteristics were recorded using a solar simulator under AM 1.5 G solar illumination; the active area of the device was 0.25 cm^2^. We determined the efficiency of five devices and averaged the data. The results are shown in Figure 4a, and the device parameters are listed in Table 2. Compared with the devices based on pure TiO_2_-NTs, the device efficiency based on TiO_2_-NTs@cut-MWCNTs improved to 0.0138%. This is because the cut-MWCNTs have better light absorption properties and promote the efficient separation of photogenerated charges.

In order to evaluate the charge transfer of the as-synthesized TiO_2_-NTs@cut-MWCNT heterostructures, the transient photocurrent response of TiO_2_-NT and TiO_2_-NTs@cut-MWCNT heterostructures was studied by using a CEL-S500/350 xenon lamp, a CHI-660E workstation, and a control system to build a simulated sunlight test device. A standard three-electrode system was used, with a Pt sheet as the counter electrode, AgCl as the reference electrode, TiO_2_-NTs or TiO_2_-NTs@cut-MWCNTs as the working electrode, and a 0.1 M Na_2_S aqueous solution as the electrolyte, under AM 1.5 G light intensity. The transient photogenerated current density test time was 400 s, and the interval between turning the light source on and off was 20 s. In Figure 4b, it can be seen that the photocurrent density of TiO_2_-NTs is 17.48 μA/cm^2^, and the photocurrent density of the TiO_2_-NTs@cut-MWCNT heterostructure is 19.94 μA/cm^2^, exhibiting a significantly enhanced transient photocurrent. The density response is due to: (i) the enhanced absorption of visible light by the TiO_2_-NTs@cut-MWCNT heterostructure generating a greater number of carriers under illumination and (ii) the enhanced separation of photogenerated carriers prolonging the electron lifetime.

This work represents the first attempt at the construction of all-titanium–carbon photovoltaic devices. There are many factors that affect the photovoltaic performance. For example, we can achieve better charge transmission by improving the contact density of cut-MWCNTs and TiO_2_-NTs so as to improve the efficiency of photovoltaic devices.

### 3.4. Mechanism of Photocurrent Generation

We calculated the band gap by the Kubelka–Munk function. Figure S4 shows the band gap of TiO_2_-NTs. The (αhν)^1/2^ of hν in the vicinity of the absorption edge is plotted for TiO_2_-NTs in Figure 3e, where hν is the photo energy, and the reflectance is converted to an equivalent absorption coefficient by α = (1 − R)^2^/2R [33]. Figure 3e shows that the light absorption range of TiO_2_-NTs is mostly in the ultraviolet region, which means that higher energy absorption is required for electronic transitions to occur; by extrapolating the linear part of the curve to the intersection with the *x*-axis, its band gap is estimated to be 3.18 eV, which is in line with the band-gap width of anatase TiO_2_ [34]. Generally, the oxidation of graphene will open the electron energy gap, and an increased degree of oxidation results in a larger band gap [35,36]. In addition, graphene oxide has been found to be a p-type or n-type semiconductor [37,38]. Therefore, the band gap of carbon nanotubes can be tuned by different degrees of oxidation to meet specific photovoltaic applications.

**Figure 5:**
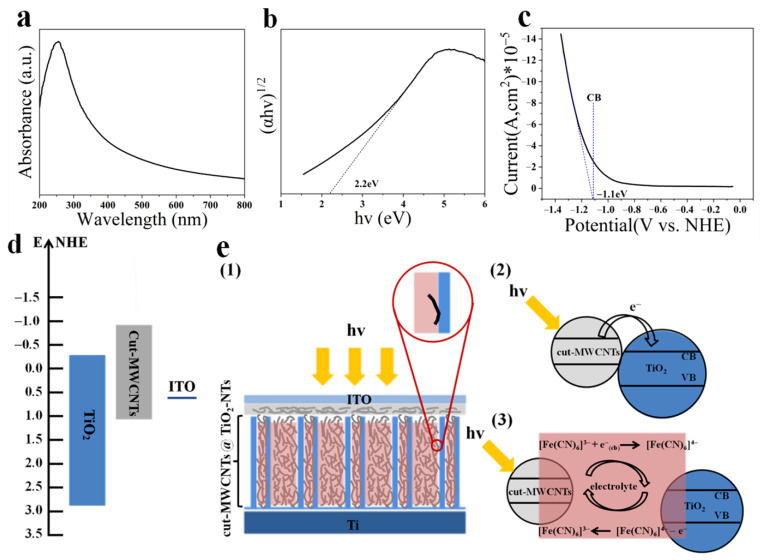
(**a**) Optical absorption spectra of cut-MWCNTs; (**b**) plots of (αhν)^1/2^ against the photon energy (hν) for cut-MWCNTs; (**c**) cathodic linear potential scan for determining the conduction band (CB) edge of the cut-MWCNTs; (**d**) schematic diagram of device energy level; (**e**) electron transport mechanisms in photovoltaic devices.

Through the optical absorption spectrum and the linear potential scan, the energy-level position of the cut-MWCNTs can be obtained. Figure 5a shows the UV-vis absorption spectrum of cut-MWCNTs. According to the Kubelka–Munk function, the forbidden bandwidth is calculated to be 2.2 eV [39], as shown in Figure 5b. Figure 5c shows that the conduction band position of cut-MWCNTs is calculated to be −1.1 eV (vs. NHE) by linear potential scanning [39]. Figure 5d shows the energy-level schematic diagram of TiO_2_ and cut-MWCNTs.

The electron transport mechanism in photovoltaic devices is shown in Figure 5e. Figure 5e (1) is a cross-sectional view of the photovoltaic device. TiO_2_ and carbon nanotubes are connected by direct contact and electrolyte contact, so we conclude that the photocurrent is generated mainly through the following two pathways: (i) Direct-contact electron conduction, as shown in Figure 5e (2). The photoexcitation of cut-MWCNTs leads to rapid electron injection into TiO_2_. (ii) Indirect-contact electron conduction, as shown in Figure 5e (3). The electrolyte acts as a “bridge”, whereby cut-MWCNTs are excited by light to generate electrons, and the oxidized electrolyte [Fe(CN)_6_]^3−^ accepts electrons and changes to the reduced state [Fe(CN)_6_]^4−^. TiO_2_ then oxidizes [Fe(CN)_6_]^4−^ to [Fe(CN)_6_]^3−^. The reactions are:

(cut-MWCNTs) + hν → (cut-MWCNTs)*(1)

(cut-MWCNTs)* → (cut-MWCNTs)^+^ + e^−^_(cb)_ (2)

[Fe(CN)_6_]^3−^ + e^−^_(cb)_ → [Fe(CN)_6_]^4−^ (3)

TiO_2_ + [Fe(CN)_6_]^4−^ → (TiO_2_)^−^ + [Fe(CN)_6_]^3−^ (4)

## 4. Conclusions

In conclusion, we prepared highly ordered TiO_2_-NTs on a metal Ti substrate by anodic oxidation. Short cut-MWCNTs were obtained by chemical oxidation. The tests and analyses showed that the graphene structure of MWCNTs was destroyed, and a large number of negatively charged oxygen-containing functional groups were introduced to the surface. Under the action of an external electric field, cut-MWCNTs were filled into TiO_2_-NTs to form the TiO_2_-NTs@cut-MWCNT heterostructure. The test results show that the two materials effectively combined. Chemically modified MWCNTs have a band gap of 2.2 eV and have a suitable energy level matching that of TiO_2_. Compared with pure TiO_2_-NTs, the TiO_2_-NTs@cut-MWCNT heterostructure exhibits higher light absorption and a higher transient photocurrent density, and the fabricated photovoltaic device efficiency increases from 0.01036% to 0.01380%.

This is a new attempt to adjust the band gap of carbon nanotubes through oxidation to achieve energy level matching with TiO_2_ and to prepare photovoltaic devices with semiconducting carbon nanotubes as photosensitizers. We hope this study provides a reference for the application of carbon nanotubes in photovoltaic devices and promotes the further development for the efficient large-scale production of all-titanium–carbon photovoltaic devices.

## Figures and Tables

**Table 1:** Elemental composition of MWCNTs and cut-MWCNTs.

Sample	C [%]	O [%]	N [%]	S [%]
MWCNTs	95.7	4.3	--	--
cut-MWCNTs	82.55	15.46	1.57	0.42

**Table 2:** Photovoltaic device parameters based on TiO_2_-NTs and TiO_2_-NTs@cut-MWCNTs.

Sample	Voc (V)	Jsc (mA/cm^2^)	FF [%]	PCE [%]
TiO_2_-NTs	0.19	0.148	0.365	0.01036
TiO_2_-NTs@cut-MWCNTs	0.22	0.164	0.381	0.01380

## Data Availability

All data, models, and code generated or used during the study appear in the submitted article.

## Supplementary Materials

The following supporting information can be downloaded at: https://www.mdpi.com/article/10.3390/nano12203604/s1, Figure S1: Schematic diagram of the electrophoresis device; Figure S2: (a) N_2_ adsorption-desorption isotherms; (b) NL-DFT pore size distributions of MWCNTs and cut-MWCNTs; Figure S3: Cross-sectional SEM image of the TiO_2_-NTs@cut-MWCNTs heterostructure; Figure S4: Kubelka-Munk function for band gap estimation of TiO_2_-NTs.

## References

[1] Lewis N.S. (2007). Toward cost-effective solar energy use. Science.

[2] Tang C.W. (1986). 2-Layer Organic Photovoltaic Cell. Appl. Phys. Lett..

[3] Oregan B., Gratzel M. (1991). A Low-Cost, High-Efficiency Solar-Cell Based on Dye-Sensitized Colloidal TiO_2_ Films. Nature.

[4] Mariotti N., Bonomo M., Fagiolari L., Barbero N., Gerbaldi C., Bella F., Barolo C. (2020). Recent advances in eco-friendly and cost-effective materials towards sustainable dye-sensitized solar cells. Green Chem..

[5] Gong D., Grimes C.A., Varghese O.K., Hu W.C., Singh R.S., Chen Z., Dickey E.C. (2001). Titanium oxide nanotube arrays prepared by anodic oxidation. J. Mater. Res..

[6] Paulose M., Shankar K., Varghese O.K., Mor G.K., Hardin B., Grimes C.A. (2006). Backside illuminated dye-sensitized solar cells based on titania nanotube array electrodes. Nanotechnology.

[7] Ito S., Ha N.L.C., Rothenberger G., Liska P., Comte P., Zakeeruddin S.M., Pechy P., Nazeeruddin M.K., Gratzel M. (2006). High-efficiency (7.2%) flexible dye-sensitized solar cells with Ti-metal substrate for nanocrystalline-TiO_2_ photoanode. Chem. Commun..

[8] Wu J.H., Xiao Y.M., Tang Q.W., Yue G.T., Lin J.M., Huang M.L., Huang Y.F., Fan L.Q., Lan Z., Yin S. (2012). A Large-Area Light-Weight Dye-Sensitized Solar Cell based on All Titanium Substrates with an Efficiency of 6.69% Outdoors. Adv. Mater..

[9] Nyein N., Tan W.K., Kawamura G., Matsuda A., Lockman Z. (2017). TiO_2_ nanotube arrays formation in fluoride/ethylene glycol electrolyte containing LiOH or KOH as photoanode for dye-sensitized solar cell. J. Photochem. Photobiol. A-Chem..

[10] He W., Qiu J.J., Zhuge F.W., Li X.M., Lee J.H., Kim Y.D., Kim H.K., Hwang Y.H. (2012). Advantages of using Ti-mesh type electrodes for flexible dye-sensitized solar cells. Nanotechnology.

[11] Zha C.Y., Shen L.M., Zhang X.Y., Wang Y.F., Korgel B.A., Gupta A., Bao N.Z. (2014). Double-Sided Brush-Shaped TiO_2_ Nanostructure Assemblies with Highly Ordered Nanowires for Dye-Sensitized Solar Cells. Acs Appl. Mater. Interfaces.

[12] Castrucci P., Scilletta C., Del Gobbo S., Scarselli M., Camilli L., Simeoni M., Delley B., Continenza A., De Crescenzi M. (2011). Light harvesting with multiwall carbon nanotube/silicon heterojunctions. Nanotechnology.

[13] Castrucci P., Tombolini F., Scarselli M., Speiser E., Del Gobbo S., Richter W., De Crescenzi M., Diociaiuti M., Gatto E., Venanzi M. (2006). Large photocurrent generation in multiwall carbon nanotubes. Appl. Phys. Lett..

[14] El Khakani M.A., Le Borgne V., Aissa B., Rosei F., Scilletta C., Speiser E., Scarselli M., Castrucci P., De Crescenzi M. (2009). Photocurrent generation in random networks of multiwall-carbon-nanotubes grown by an “all-laser” process. Appl. Phys. Lett..

[15] Scarselli M., Scilletta C., Tombolini F., Castrucci P., De Crescenzi M., Diociaiuti M., Casciardi S., Gatto E., Venanzi M. (2009). Photon harvesting with multi wall carbon nanotubes. Superlattices Microstruct..

[16] Scarselli M., Scilletta C., Tombolini F., Castrucci P., Diociaiuti M., Casciardi S., Gatto E., Venanzi M., De Crescenzi M. (2009). Multiwall Carbon Nanotubes Decorated with Copper Nanoparticles: Effect on the Photocurrent Response. J. Phys. Chem. C.

[17] Sarker B.K., Arif M., Stokes P., Khondaker S.I. (2009). Diffusion mediated photoconduction in multiwalled carbon nanotube films. J. Appl. Phys..

[18] Ou Y., Lin J.D., Fang S.M., Liao D.W. (2006). MWNT-TiO_2_: Ni composite catalyst: A new class of catalyst for photocatalytic H-2 evolution from water under visible light illumination. Chem. Phys. Lett..

[19] Kierkowicz M., Pach E., Santidrian A., Sandoval S., Goncalves G., Tobias-Rossell E., Kalbac M., Ballesteros B., Tobias G. (2018). Comparative study of shortening and cutting strategies of single-walled and multi-walled carbon nanotubes assessed by scanning electron microscopy. Carbon.

[20] Kumar S.S., Vairam S., Neelakandeswari N., Aruna S. (2018). Effect of metal oxide charge transfer layers on the photovoltaic performance of carbon nanotube heterojunction solar cells. Mater. Lett..

[21] Kalita G., Adhikari S., Aryal H.R., Umeno M., Afre R., Soga T., Sharon M. (2008). Cutting carbon nanotubes for solar cell application. Appl. Phys. Lett..

[22] Baker B.A., Zhang H., Cha T.G., Choi J.H., Yamashita S., Saito Y., Choi J.H. (2013). 9—Carbon nanotube solar cells. Carbon Nanotubes and Graphene for Photonic Applications.

[23] Macak J.M., Gong B.G., Hueppe M., Schmuki P. (2007). Filling of TiO_2_ Nanotubes by Self-Doping and Electrodeposition. Adv. Mater..

[24] Hoffmann M.R., Martin S.T., Choi W., Bahnemann D.W. (1995). Environmental Applications of Semiconductor Photocatalysis. Chem. Rev..

[25] Wang W., Serp P., Kalck P., Faria J. (2005). Visible Light Photodegradation of Phenol on MWNT-TiO_2_ Composite Catalysts Prepared by a Modified Sol–Gel Method. J. Mol. Catal. A Chem..

[26] Banerjee S., Wong S. (2002). Synthesis and Characterization of Carbon Nanotube−Nanocrystal Heterostructures. Nano Lett.-NANO LETT.

[27] Su B.Z., Wang S.C., Yang W.B., Wang Y., Huang L.J., Popat K.C., Kipper M.J., Belfiore L.A., Tang J.G. (2020). Synthesis of Eu-modified luminescent Titania nanotube arrays and effect of voltage on morphological, structural and spectroscopic properties. Mater. Sci. Semicond. Process..

[28] Dai H.J. (2002). Carbon nanotubes: Opportunities and challenges. Surf. Sci..

[29] Song Y.P., Feng M., Zhan H.B. (2014). Electrochemistry of partially unzipped carbon nanotubes. Electrochem. Commun..

[30] Saito T., Matsushige K., Tanaka K. (2002). Chemical treatment and modification of multi-walled carbon nanotubes. Phys. B.

[31] Li F., Wang Y., Wang D., Wei F. (2004). Characterization of single-wall carbon nanotubes by N_2_ adsorption. Carbon.

[32] Iijima S. (1991). Helical Microtubules of Graphitic Carbon. Nature.

[33] Reyes-Coronado D., Rodríguez-Gattorno G., Espinosa-Pesqueira M.E., Cab C., de Coss R., Oskam G. (2008). Phase-pure TiO_2_nanoparticles: Anatase, brookite and rutile. Nanotechnology.

[34] Gong J.W., Liang J., Sumathy K. (2012). Review on dye-sensitized solar cells (DSSCs): Fundamental concepts and novel materials. Renew. Sust. Energ. Rev..

[35] Boukhvalov D.W., Katsnelson M.I. (2008). Modeling of graphite oxide. J. Am. Chem. Soc..

[36] Wang D.H., Choi D.W., Li J., Yang Z.G., Nie Z.M., Kou R., Hu D.H., Wang C.M., Saraf L.V., Zhang J.G. (2009). Self-Assembled TiO_2_-Graphene Hybrid Nanostructures for Enhanced Li-Ion Insertion. ACS Nano.

[37] Wu X.S., Sprinkle M., Li X.B., Ming F., Berger C., de Heer W.A. (2008). Epitaxial-graphene/graphene-oxide junction: An essential step towards epitaxial graphene electronics. Phys. Rev. Lett..

[38] Gilje S., Han S., Wang M., Wang K.L., Kaner R.B. (2007). A chemical route to graphene for device applications. Nano Lett..

[39] Yeh T.F., Chan F.F., Hsieh C.T., Teng H.S. (2011). Graphite Oxide with Different Oxygenated Levels for Hydrogen and Oxygen Production from Water under Illumination: The Band Positions of Graphite Oxide. J. Phys. Chem. C.

