# Supplementary material for: Heterostructures of Cut Carbon Nanotube-Filled Array of TiO2 Nanotubes for New Module of Photovoltaic Devices
Source: Nanomaterials (Basel). 2022 Oct 14;12(20):3604. doi: 10.3390/nano12203604 (PMC9608834; doi:10.3390/nano12203604)
Supplement: Supplementary file 1 [file nanomaterials-12-03604-s001.zip › nanomaterials-1885385-supplementary.pdf]

Supplementary Materials

Heterostructures of Cut Carbon Nanotube-Filled Array of TiO_2 Nanotubes for New Module of Photovoltaic Devices

Siqi Niu [1], Wenbin Yang [1], Heng Wei [1], Michail Danilov [2], Ihor Rusetskyi [2], Ketul C. Popat [3,4,*], Yao Wang [1], Matt J. Kipper [3,4], Laurence A. Belfiore [1,4] and Jianguo Tang [1,*]

[1] Institute of Hybrid Materials, National Center of International Joint Research for Hybrid Materials Technology, National Base of International Sci. & Tech. Cooperation on Hybrid Materials, Qingdao University, 308 Ningxia Road, Qingdao 266071, China
[2] V.I. Vernadskii Institute of General and Inorganic Chemistry of the Ukrainian NAS, 32/34 Palladin avenue, 03142 Kyiv, Ukraine
[3] Department of Mechanical Engineering, Colorado State University, Fort Collins, CO 80523, USA
[4] Department of Chemical and Biological Engineering, Colorado State University, Fort Collins, CO 80523, USA
* Correspondence: ketul.popat@colostate.edu (K.C.P.); tang@qdu.edu.cn (J.T.)

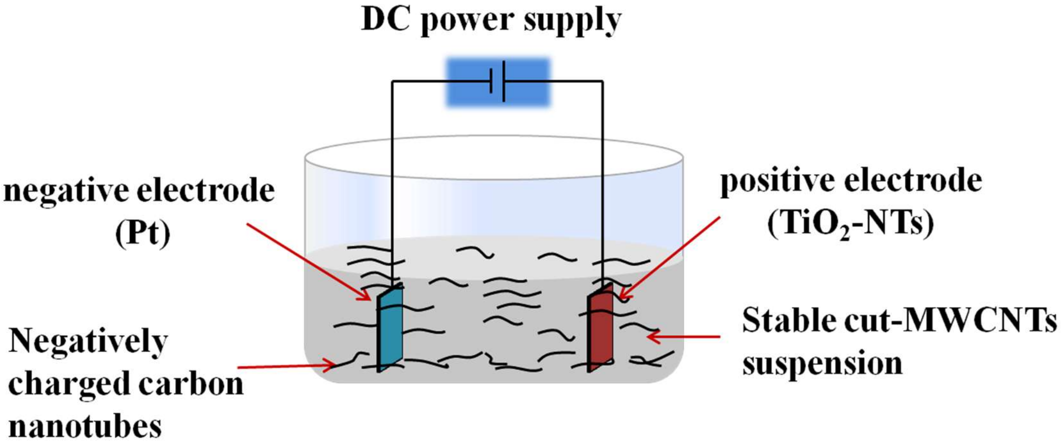

Figure S1. Schematic diagram of the electrophoresis device.

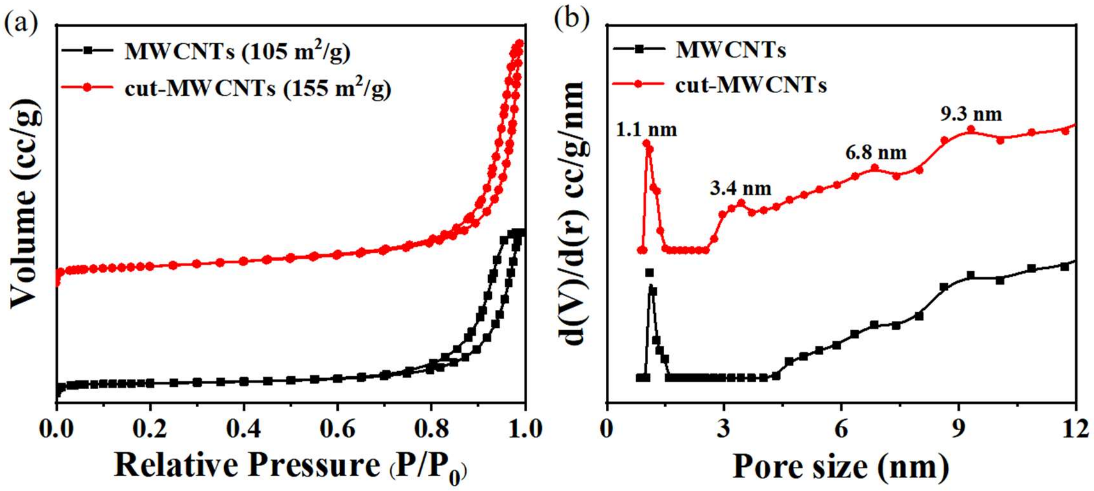

Figure S2. (a) N_2 adsorption-desorption isotherms; (b) NL-DFT pore size distributions of MWCNTs and cut-MWCNTs.

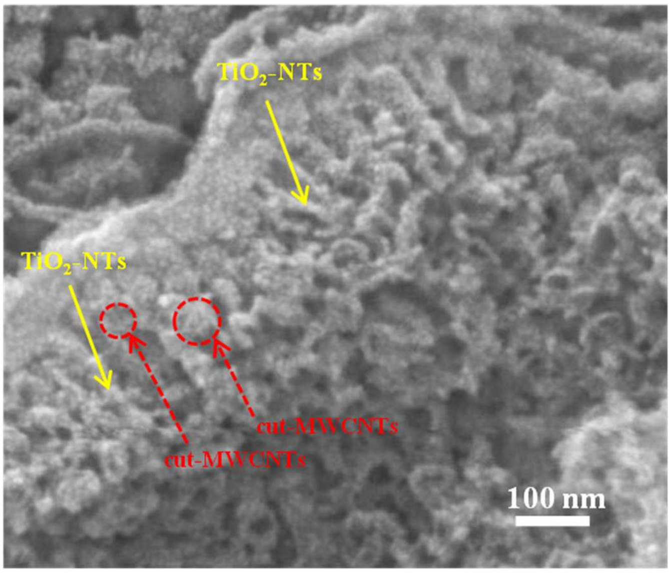

Figure S3. Cross-sectional SEM image of the TiO_2-NTs@cut-MWCNTs heterostructure.

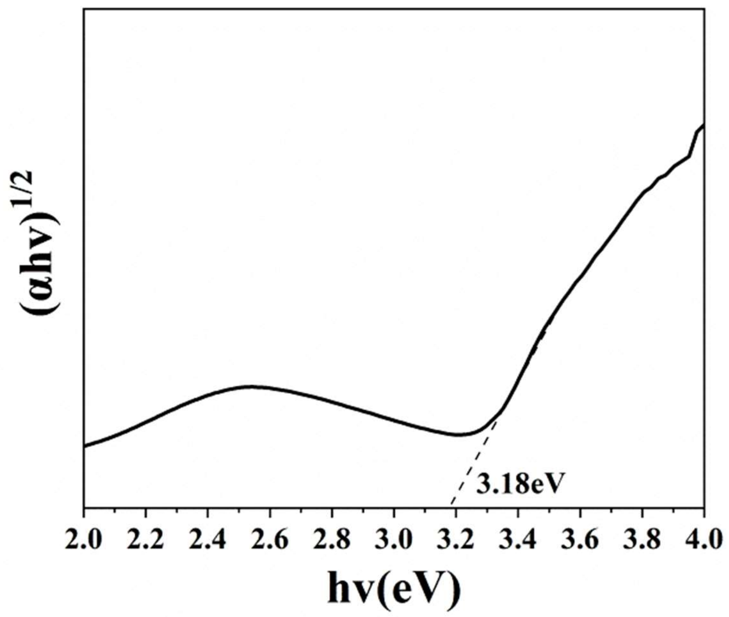

Figure S4. Kubelka-Munk function for band gap estimation of TiO_2-NTs.
